# Machine learning-based automated classification of headache disorders using patient-reported questionnaires

**DOI:** 10.1038/s41598-020-70992-1

**Published:** 2020-08-20

**Authors:** Junmo Kwon, Hyebin Lee, Soohyun Cho, Chin-Sang Chung, Mi Ji Lee, Hyunjin Park

**Affiliations:** 1 Department of Electrical and Computer Engineering, Sungkyunkwan University, Suwon, 16419 South Korea; 2 Center for Neuroscience Imaging Research, Institute for Basic Science, Suwon, 16419 South Korea; 3 Department of Neurology, Neuroscience Center, Samsung Medical Center, Sungkyunkwan University School of Medicine, Seoul, 06351 South Korea; 4 School of Electronic and Electrical Engineering, Center for Neuroscience Imaging Research, Sungkyunkwan University, Suwon, 16419 South Korea

**Keywords:** Headache, Information technology

## Abstract

Classification of headache disorders is dependent on a subjective self-report from patients and its interpretation by physicians. We aimed to apply objective data-driven machine learning approaches to analyze patient-reported symptoms and test the feasibility of the automated classification of headache disorders. The self-report data of 2162 patients were analyzed. Headache disorders were merged into five major entities. The patients were divided into training (n = 1286) and test (n = 876) cohorts. We trained a stacked classifier model with four layers of XGBoost classifiers. The first layer classified between migraine and others, the second layer classified between tension-type headache (TTH) and others, and the third layer classified between trigeminal autonomic cephalalgia (TAC) and others, and the fourth layer classified between epicranial and thunderclap headaches. Each layer selected different features from the self-reports by using least absolute shrinkage and selection operator. In the test cohort, our stacked classifier obtained accuracy of 81%, sensitivity of 88%, 69%, 65%, 53%, and 51%, and specificity of 95%, 55%, 46%, 48%, and 51% for migraine, TTH, TAC, epicranial headache, and thunderclap headaches, respectively. We showed that a machine-learning based approach is applicable in analyzing patient-reported questionnaires. Our result could serve as a baseline for future studies in headache research.

## Introduction

Headache disorders are the most common neurological symptoms and have a substantial impact on sufferers. A proper diagnosis of headaches is essential for its treatment. Currently, the diagnosis of headache disorders is highly dependent on self-report from patients and the interpretation of the self-report by clinicians. The International Classification of Headache Disorder (ICHD) was published to aid a standardized diagnosis of headache disorders^1^. The ICHD has three chapters including primary headache disorders, secondary headache disorders, and painful cranial neuralgias/facial pain. These chapters include a number of disorders and their subtypes. However, its clinical application may be challenging for physicians who are inexperienced in headache medicine.

There have been efforts to aid the diagnosis of primary headache disorders using neurophysiological tests^2^, neuroimaging^3,4^, and blood-based biomarkers^5,6^; however, these have not replaced clinical interviews. Previous studies have mainly focused on migraine with little focus on the differential diagnosis of other headache disorders^7,8^. Recently, a simple questionnaire for the screening of migraine was developed and validated for research purposes^9^. The clinical diagnosis of headache disorders should, however, be based on a holistic approach since a single characteristic cannot replace the proper diagnosis.

Recently, data-driven approaches using machine learning or deep learning have been tested in the medical field to avoid biases attributed to human factor^10–12^. These approaches have been used mostly for neuroimaging analysis in headache research^3,4^. In this study, we aimed to analyze self-reported symptoms of patients to classify four headache disorders including migraine, by using machine learning approaches. Real-world questionnaires obtained from more than 2000 patients were used for this study.

## Methods

### Subjects

This study was approved by the institutional review board (IRB) of the Samsung Medical Center (IRB 2018-10-029). Written informed consents were obtained from patients or their guardians. Our study was performed in full accordance with local IRB guidelines. A total of 2162 patients who visited our headache clinic for the first time between January 2017 and December 2018 were included in our prospective headache clinic registry. The registry was retrospectively screened for this study. All patients completed structured questionnaires. Based on the questionnaire and clinical interview, the diagnosis of headache disorders was made using the ICHD-3 beta or ICHD-3 (whichever was the most updated at the time of visit) by headache specialists (MJL with 10 years of experience and C-SC with 30 years of experience).

### Headache clinic registry

The questionnaire for assessing patients on their first visit was developed by a headache specialist (MJL) and has been used in the Samsung Medical Center headache clinic since 2015. The questionnaire consists of 75 screening questions (Supplementary Table S1) including headache characteristics (e.g., intensity, location, nature of pain, and aggravation during or avoidance of physical activities), disease course (onset, the mode of onset, and the time of aggravation), associated symptoms (e.g., nausea, vomiting, photophobia, phonophobia, osmophobia, autonomic symptoms), aura, information regarding the medication used for headaches, past medical history (e.g. hypertension, diabetes, insomnia, depression, anxiety, and others), and social history (e.g. caffeine intake, smoking, alcohol consumption, and occupation). In addition to data from the questionnaire, patient demographics including age, sex, and body mass index (BMI) were prospectively recorded in the headache clinic registry and used for the analysis in this study. The ICHD-3-based diagnosis of each patient was coded in the registry.

The data of 2018 were used as the training cohort and the data of 2017 were used as the test cohort. There was no overlap between the two cohorts because the questionnaire was evaluated only for new patients. For the analysis, we merged headache disorders with similar entities into seven groups: migraine, tension-type headache (TTH), trigeminal autonomic cephalalgia (TAC), epicranial headache (including primary stabbing headache and occipital neuralgia), thunderclap headache ([TCH] including primary and secondary causes of TCH), other primary headache disorders, and secondary headaches other than those causing TCHs. Among these, we excluded other primary headaches (n = 49, training cohort) due to the high heterogeneity in the subtype. Secondary headache disorders other than TCH (n = 122, training cohort) were also excluded because this subtype presented with heterogeneous diseases that required diagnoses from the clinical course rather than headache characteristics. Data from 2162 patients, including the training cohort (n = 1286) and test cohort (n = 876), were finally used for this study. Further details of the patients are given in Table 1.

**Table 1:** Distribution of primary headache subtypes.

Information	Training cohort (n = 1,286)	Test cohort (n = 876)	*p* value
**Age**			
Mean (SD)	47 (15)	45 (15)	0.1092
Range (IQR)	11–90 (35–57)	14–88 (34–56)	
**Sex**			
male:female	373:913	275:601	0.2534
**Subtypes**			
Migraine	864	600	0.8329
Tension-type headache	144	91	
Trigeminal autonomic cephalalgia	79	57	
Epicranial headache	104	61	
Thunderclap headache	95	67	

Values are reported as mean with standard deviation in parenthesis. *p *values were obtained from the Kolmogorov–Smirnov test for continuous information and Chi-square test for categorical information.

*SD* standard deviation, *IQR* inter-quartile range.

### Stacked classifier model

**Figure 1:**
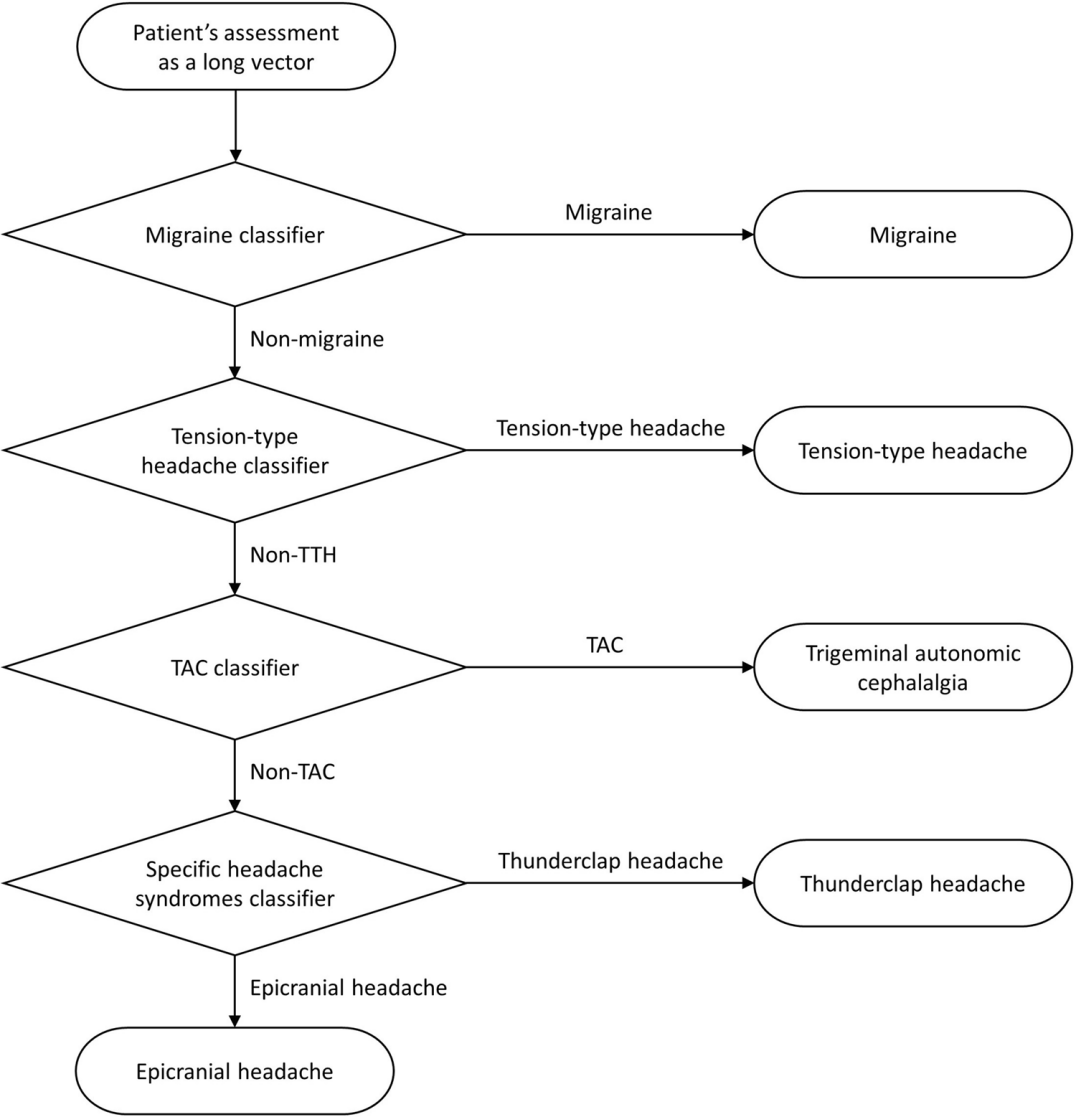
Structure of the stacked classifier model. *TTH* tension-type headache, *TAC* trigeminal autonomic cephalalgia.

We adopted a stacked model that consisted of four layers of binary XGBoost^13^ classifiers as shown in Fig. 1. Each layer of binary XGBoost classifier was used to classify subjects into two groups: the target subtype and the rest. We explored all possible orders of the stacked classifier and chose the order with the best accuracy in the training cohort. The first layer classified the most dominant subtype (i.e., migraine) and the rest (i.e., non-migraine). This enabled less challenging issues to be tackled first. The second layer classified between TTH and the rest (i.e., non-TTH). The third layer classified TAC and the rest (i.e., epicranial headaches and TCH). The final layer classified epicranial headaches and TCH. Our ordering of the classifier is similar to a multi-scale approach where one starts solving a large-scale problem before progressing on to small-scale problems.

### Feature selection

Each patient assessment was turned into a long feature vector. Continuous variable responses were normalized with a value between − 1 and 1. Categorical variable responses were converted to a one-hot vector. Multi-hot encoding was adopted for some categorical questions with multiple responses. Thus, the assessment of 75 questions for each patient was transformed into features with 128 dimensions. We applied the least absolute shrinkage and selection operator (LASSO)^14^ in choosing a few important features for each stacked classifier layer. For example, the LASSO was used to select features that can distinguish between migraine from non-migraine subtypes in the first layer. The LASSO was applied using the stratified tenfold cross-validation. From the cross-validation, features that appeared at least three times out of the ten folds were chosen. These features were chosen as the set of stable features and the threshold of three was chosen to maximize the classifier performance on average in the left-out fold in the training cohort within the tenfold cross-validation. The final model was re-trained using the stable features from the entire training cohort.

### Classifier performance

The selected stable features were used to train the stacked XGBoost classifier. The trained classifier was evaluated on the independent test cohort. Sensitivity, specificity, and accuracy were assessed to quantify the performance of the classifiers in both cohorts. The classifiers were also evaluated using minimum sensitivity and specificity among the subtypes, to provide summary statistics over the subtypes. A confusion matrix was also provided.

### Comparison with other methods

To ensure the methods used in our study are well-suited in classifying headache subtypes, we compared our feature selection method (LASSO) with support vector machine recursive feature elimination (SVM-RFE)^15^ and minimum-redundancy maximum-relevancy (mRMR)^16^ approaches. The numbers of the selected features using mRMR and SVM-RFE for each classifier layer were fixed as those of LASSO. The selected features were fed into the stacked XGBoost classifier. We also compared XGBoost with other binary classifiers such as k-nearest neighbor (k-NN), support vector machine (SVM), and random forest in each of the stacked layers with features selected by LASSO.

## Results

### Selected features

The feature selection procedure led to 32, 19, 6, and 22 features that corresponded to the first, second, third, and fourth layers of the stacked classifier from the training cohort (Table 2), respectively. Table 2 showed selected features positively correlated with the corresponding target subtypes. The top three prominent features in the first layer (migraine vs. non-migraine) were mode of onset: gradual, female sex, and absence of lacrimation. The top three prominent features in the second layer (TTH vs. non-TTH) were mode of onset: gradual, nature of pain: vague/cloudy, and cognitive complaint during headache attack. The top three prominent features in the third layer (TAC vs. specific headache syndromes including epicranial headache and thunderclap headache) were headache attack during sleep, headache triggered by upset stomach, and conjunctival injection. The top three prominent features in the fourth layer (epicranial headache vs. thunderclap headache) were location: retroauricular, nature of pain: electric shock-like, and nature of pain: jabbing, assuming epicranial headache as the positive subtype in the specific headache syndromes classifier.

**Table 2:** Selected features from different layers of the classifier.

Layer	Selected features
First (migraine classifier) 32 features	Mode of onset: gradual (1st), female sex (2nd), absence of lacrimation (3rd), nausea/vomiting, headache triggered by upset stomach, not located in the temple, photophobia, absence of conjunctival injection, absence of brainstem aura: vertigo, ear fullness/tinnitus, not located in the vertex, headache-related disability in daily routines, absence of headache attack during sleep, aggravation by physical activity, not in location: all over the head, not in location: back of the head, osmophobia, not in location: retroauricular, head motion-induced worsening, phonophobia, no pulsating nature, throbbing nature, no stabbing nature, absence of motion sickness, absence of agitation, vertigo, headache-associated ocular pain, nature of pain: vague/cloudy, dull-ache-like nature, general weakness, not in location: forehead, dizziness
Second (TTH classifier) 19 features	Mode of onset: gradual (1st), nature of pain: vague/cloudy (2nd), cognitive complaint during headache attack (3rd), hypertension, absence of head motion-induced worsening, absence of avoidance of physical activity, nature of pain: dull-ache, no jabbing nature, nature of pain: drumming, absence of headache-induced awakening during sleep, nature of pain: pulsating, nausea/vomiting, headache attack in the afternoon, absence of headache-associated ocular pain, absence of aggravation by physical activity, absence of ocular pain, absence of disability in daily routines, female sex
Third (TAC classifier) 6 features	Headache attack during sleep (1st), headache triggered by upset stomach (2nd), conjunctival injection (3rd), location: periocular, lacrimation, male sex
Fourth (Epicranial headache classifier) 22 features	Location: retroauricular (1st), nature of pain: electric shock-like (2nd), nature of pain: jabbing (3rd), absence of headache triggered by upset stomach, no explosive nature, no tightening nature, no drumming nature, mode of onset: gradual, allodynia, nature of pain: tingling, absence of alleviation by sleeping, nature of pain: stabbing, location: temple, absence of aggravation by physical activity, nature of pain: vague/cloudy, no dull-ache-like nature, absence of ocular pain, photophobia, absence of nausea/vomiting, nature of pain: twinge, absence of headache-associated gastrointestinal discomfort
Fourth (TCH classifier) 22 features	Not in location: retroauricular (1st), no electric shock-like nature (2nd), no jabbing nature (3rd), headache triggered by upset stomach, nature of pain: explosive, nature of pain: tightening, nature of pain: drumming, mode of onset: thunderclap, absence of allodynia, no tingling nature, alleviation by sleeping, no stabbing nature, not in location: temple, aggravation by physical activity, no vague/cloudy nature, nature of pain: dull-ache, ocular pain, absence of photophobia, nausea/vomiting, no twinge nature, headache-associated gastrointestinal discomfort

The features listed in the right column positively correlated with the target subtype listed in the left column.

*TTH* tension-type headache, *TAC* trigeminal autonomic cephalalgia, *TCH* thunderclap headache.

### Classifier performances

The performances of the classifiers for both cohorts were given in Table 3 and the performance using the confusion matrices were in Tables 4 and 5. The stacked XGBoost classifier using the selected features attained an accuracy of 82%, sensitivity of 87%, 66%, 85%, 65%, and 64% for the five subtypes, and specificity of 94%, 54%, 58%, 63%, and 57% for the five subtypes in the training cohort. The baseline accuracy (i.e., assigning all cases as the dominant subtype) was 67%. The stacked XGBoost classifier using the selected features led to an accuracy of 81%, sensitivity of 88%, 69%, 65%, 53%, and 51% for the five subtypes, and specificity of 95%, 55%, 46%, 48%, and 51% for the five subtypes in the test cohort. The baseline accuracy (i.e., assigning all cases as the dominant subtype) was 68%. Our approach performed better (*p* value < 10^−8^) than the baseline naïve classifier in the test cohort using Fisher’s exact test.

**Table 3:** Classifier performance of both cohorts.

Cohort	Baseline (%)	Accuracy (%)	Headache subtype	Sensitivity (%)	Specificity (%)
Training	67.19	81.80	Migraine	87.07	93.52
			Tension-type headache	66.10	54.17
			Trigeminal autonomic cephalalgia	85.19	58.23
			Epicranial headache	64.71	63.46
			Thunderclap headache	64.29	56.84
Test	68.49	80.71	Migraine	88.47	94.67
			Tension-type headache	69.44	54.95
			Trigeminal autonomic cephalalgia	65.00	45.61
			Epicranial headache	52.73	47.54
			Thunderclap headache	50.75	50.75

**Table 4:** Confusion matrix for the training cohort. The bold numbers in the main diagonal denote correctly classified subjects.

Headache subtype	Migraine	Tension-type headache	Trigeminal autonomic cephalalgia	Epicranial headache	Thunderclap headache
Migraine	**808**	23	3	13	17
Tension-type headache	46	**78**	1	13	6
Trigeminal autonomic cephalalgia	18	4	**46**	6	5
Epicranial headache	25	9	2	**66**	2
Thunderclap headache	31	4	2	4	**54**

**Table 5:** Confusion matrix for the test cohort. The bold numbers in the main diagonal denote correctly classified subjects.

Headache subtype	Migraine	Tension-type headache	Trigeminal autonomic cephalalgia	Epicranial headache	Thunderclap headache
Migraine	**568**	5	4	7	16
Tension-type headache	25	**50**	1	9	6
Trigeminal autonomic cephalalgia	21	2	**26**	2	6
Epicranial headache	11	13	3	**29**	5
Thunderclap headache	17	2	6	8	**34**

### Comparison of feature selection methods

Our feature selection method (LASSO) was compared to SVM-RFE and mRMR approaches. The numbers of the selected features using mRMR and SVM-RFE for each classifier layer were fixed as those of LASSO. The selected features were fed into the stacked XGBoost classifier. An overall accuracy, minimum sensitivity, and minimum specificity of the stacked XGBoost classifier were evaluated in the test cohort. Table 6 showed that features obtained through LASSO led to the best performance in the test cohort.

**Table 6:** Comparison of the proposed method with other feature selection methods in the test cohort in terms of classifier performance. The bold values indicate the highest score in each performance metric.

Feature selection method	Accuracy	Minimum sensitivity	Minimum specificity
LASSO	**0.8071**	**0.5273**	**0.4561**
SVM-RFE	0.8014	0.4468	0.3443
mRMR-MIQ	0.7180	0.1600	0.0877
mRMR-MID	0.7055	0.0833	0.0597

*LASSO* least absolute shrinkage and selection operator, *SVM-RFE* support vector machine recursive feature elimination, *mRMR-MIQ* minimum-redundancy maximum-relevancy mutual information quotient, *mRMR-MID* minimum-redundancy maximum-relevancy mutual information difference.

### Comparison of binary classifiers

We compared XGBoost with k-NN, SVM, and random forest classifiers in each of the stacked layers in terms of overall accuracy, minimum sensitivity, and minimum specificity. The evaluation was performed in the test cohort (Table 7). The same features chosen from the feature selection stage using LASSO were used for all the classifiers. Although there were small differences in classifier performances, XGBoost still outperformed the other classifiers.

**Table 7:** Comparison of the proposed method with other classifiers in the test cohort. The bold values indicate the highest score in each performance metric.

Classifier	Accuracy	Minimum sensitivity	Minimum specificity
XGBoost	**0.8071**	**0.5273**	**0.4561**
Random forest	0.8037	0.5179	0.4035
SVM	0.7911	0.4730	0.4035
k-NN	0.7717	0.4355	0.3333

*SVM* support vector machine, *k-NN* k-nearest neighbor.

## Discussion

In this study, we applied a machine learning approach to classify major headache disorders using questionnaires completed by patients in a real-world setting. We found that machine learning is applicable in analyzing questionnaires. The performance of the machine learning approach in the classification of migraine was excellent however, its accuracy in classifying headache disorders other than migraine was inferior to that in classifying migraine. Nonetheless, our automated classification results could be still meaningful as the gold standard for the diagnosis of headache is a manual skillful application of the current classification criteria (currently ICHD-3, published in 2018). In the era of ICHD-3, there have been no studies evaluating the reliability and accuracy of the diagnosis of primary headache disorders made by primary care providers or general non-headache neurologists. Furthermore, there has been no classification methods other than ICHD-3.

Our study is one of the first studies to apply machine learning in the analysis of patient-reported questionnaires to classify primary headache disorders^7^. The diagnosis of headache disorders requires a skillful interview with patients and a comprehensive decision algorithm. We tested whether machine learning can substitute the role of the clinical interview. However, the samples of each headache disorder other than migraine and TTH were insufficient for the training. Headache disorders or syndromes other than migraine and TTH were merged into broader categories such as epicranial headaches or TCHs, which was not ideal for the detailed classification of second- or third-digit ICHD codes. In addition, secondary headaches other than those causing TCHs were excluded from the analysis since they cannot be incorporated into one entity. Secondary headaches should be diagnosed by clinical courses and causative workups rather than headache features. Taken together, our approach could not replace physician-based diagnosis due to insufficient results. However, this study demonstrated the feasibility of developing a better algorithm-based automated classification for headache disorders. Besides, our results might be used to inform or assist physicians by pre-screening with the most important factors of the stacked classifier (i.e., Table 2) or increasing the accuracy of less-specialized providers.

Our approach adopted a stacked XGBoost classifier that resulted in an overall accuracy of 81%, sensitivity and specificity of over 87% in the diagnosis of migraine. Our results were superior to the results from a previous study in which more selective data were used^7^. Existing studies on the classification of headache disorders with machine learning have focused on a few selected headache disorders such as migraine and tension-type headache due to challenges with sample size^7,8^. Previous studies used the random forest for classification however, our study adopted the XGBoost. XGBoost belongs to the boosting classifiers in which both the variance and bias of the classifier is reduced, while random forest belongs to the bagging classifiers in which only the variance of the classifier is reduced^13^. XGBoost has shown improved performance in many recent machine learning challenges where high-dimensional features were involved. The performance of XGBoost in classifying migraine was superior in our study because migraine is characterized by diverse features which cannot be fully incorporated in conventional statistical models, due to the complexity and challenge of multiple testing. Manual analysis even by human experts, may be time-consuming and prone to errors. However, with the automated classification algorithm suggested by this study, multiple features of headache disorders can be systematically identified. This automated classification algorithm is thus time efficient and could minimize human error in the diagnosis of headache disorders.

Our stacked classification model well reflected features of each headache disorder. Top three features used in our classification model show insights into each headache disorder when compared to the ICHD-3 criteria^1^. First, the mode of onset was important in migraine, TTH, and epicranial vs. TCH classifiers. This important feature should be always considered in the differential diagnosis of secondary and primary headaches, but it has not been listed in the ICHD-3 criteria for migraine, TTH, and epicranial headaches^1^. While migraine and TTH are typical examples of gradual-onset headaches, thunderclap onset is the most important syndrome-defining features of TCH as its nomenclature implies. For TAC, the mode of onset was not included in the classifier, as most patients with TAC experiences a relatively rapid evolution of headache attack. Second, the demographic feature was also important, while the ICHD only deals with headache characteristics. For example, female sex was ranked as the second important feature of classifying migraine. This may suggest that the female predominance is more robust in migraine than in other primary headache disorders at least in clinic-based samples. Third, the nature of pain was important in TTH and epicranial vs. TCH classifiers: vague and/or cloudy nature of pain for TTH and electric-shock like and jabbing natures for epicranial headaches. These features well reflect the nature of corresponding headache disorders, although they are different from features listed in ICHD-3 criteria^1^. The ICHD-3 denotes pressing or tightening quality of pain as features of TTH and stabbing, shooting, or sharp quality of pain as epicranial (primary stabbing headache or occipital neuralgia) headaches^1^. However, these features may be less useful in the differential diagnosis as they can co-exist in migraine attacks, TACs, and even TCHs in the real world. Fourth, the presence or absence of autonomic symptoms was important in differentiating migraine and TACs. The ICHD-3 also denotes autonomic symptoms as characteristic features of TAC^1^. Although autonomic symptoms can accompany migraine attacks, they are less prominent when compared to those of TACs^17^. Finally, sleep-awakening hypnic attacks were important in the TAC classifier. The time of headache attack has not been included in the ICHD-3. However, most of the primary headaches other than TAC tend to regress during sleep. In summary, our data showed these features can have greater relative weights in the differential diagnosis between primary headache disorders even though they are not listed as or different from syndrome-defining features in the ICHD-3^1^.

To apply our study results to clinical practice, it should be kept in mind that secondary headache disorders were excluded in this model. This may have some clinical implications: in addition to clinical history, biochemical, radiological, or sometimes histologic evaluations are needed to rule out secondary headache syndromes. Historically, the clinical course rather than headache characteristics has been more important, whilst this cannot be easily captured by the questionnaire. Still, we explored whether automated classification was possible using the same approach. The classification performance was unsatisfactory as shown in the Supplement.

Our study has some limitations. First, the results were derived from data from a single center. Thus, our results need to be validated in an independent cohort study. Second, we applied conventional machine learning approaches in this study. Deep learning could be thought of as a high degree-of-freedom extension of conventional machine learning which has significantly improved classification performance in many domains^11,18^. Deep learning could be certainly applied in headache research and we believe the autoencoder network could be effective. Autoencoder network is capable of handling high-dimensional features that are correlated and can also learn low-dimensional feature embedding that is robust to noise. The features used in headache were high-dimensional (e.g., 128 dimensions) and could have a substantial correlation among them due to how the features were designed in this study. We plan to pursue research in this direction in the future.

We presented a method to classify subtypes of primary headache by fusing four XGBoost classifiers in a stacked fashion. Each classifier captured important characteristics for the target subtype in a data-driven approach. Existing studies were insufficient as they only considered fewer subtypes and reported worse classification performance than ours. Thus, although our approach was effective for the migraine subtype only, we believe our study is a first step towards building a comprehensive computer-aided diagnosis model for headaches. The software code for this study is open and can be adopted by other researchers to foster novel machine learning research in the migraine field.

## Supplementary information

Supplementary Information 1.

## Data Availability

The data from the Samsung Medical Center is unavailable to the public due to IRB restrictions. Interested researchers should contact Dr. Mi Ji Lee (mirony.lee@gmail.com), who oversaw the data collection.

## Code availability

The code of this study is available at https://github.com/junmokwon/automated-headache-classification.
