# Supplementary material for: Machine learning-based automated classification of headache disorders using patient-reported questionnaires
Source: Sci Rep. 2020 Aug 20;10:14062. doi: 10.1038/s41598-020-70992-1 (PMC7441379; doi:10.1038/s41598-020-70992-1)
Supplement: Supplementary file 1 — Supplementary Information 1. [file 41598_2020_70992_MOESM1_ESM.docx]

**Supplementary Information**

**Title: Machine learning-based automated classification of headache disorders using patient-reported questionnaires**

Authors: Junmo Kwon^1,2^, Hyebin Lee^1,2^, Soohyun Cho^3^, Chin-Sang Chung^3^, Mi Ji Lee^3^*, and Hyunjin Park^2,4^*

^1^Department of Electrical and Computer Engineering, Sungkyunkwan University, Suwon, 16419, South Korea

^2^Center for Neuroscience Imaging Research, Institute for Basic Science (IBS), Suwon, 16419, South Korea

^3^Department of Neurology, Neuroscience Center, Samsung Medical Center, Sungkyunkwan University School of Medicine, Seoul, 06351, South Korea

^4^School of Electronic and Electrical Engineering, Sungkyunkwan University, Suwon, 16419, South Korea

*Co-corresponding authors

Mi Ji Lee, M.D. Ph.D.

Department of Neurology, Samsung Medical Center

Sungkyunkwan University School of Medicine, Seoul, 06351, South Korea

Phone: +82-2-3410-3599, Fax: +82-2-3410-1430, Email: mijilee.md@gmail.com

Hyunjin Park, Ph.D.

School of Electronic and Electrical Engineering,

Center for Neuroscience Imaging Research,

Sungkyunkwan University, Suwon, 16419, South Korea

Phone: +82-31-299-4956, Fax: +82-31-290-5819, Email: hyunjinp@skku.edu

**Supplementary Table S1.** The 75 questions used in our study.

| Index | Name of the feature | Question asked |
| --- | --- | --- |
| 1 | Age | Age |
| 2 | Sex | Gender |
| 3 | Headache-associated gastrointestinal discomfort | Do you feel discomfort in your stomach when you have a headache? |
| 4 | Headache triggered by upset stomach | Do you feel a headache when your stomach is upset? |
| 5 | Headache-related disability in daily routines | Have you experienced any disability in your daily routines such as housework, study, or work because of a headache? |
| 6 | Motion sickness | Do you have motion sickness? |
| 7 | Headache-related light sensitivity | Do you prefer a dark space when you have a headache? |
| 8 | Dizziness | Do you feel dizzy when you have a headache? |
| 9 | Alleviation by sleeping | Does your headache remit when you get some sleep? |
| 10 | Headache-associated ocular pain | Do you feel ocular pain when you have a headache? |
| 11 | Headache-induced awakening during sleep | Do you wake up because of a headache? |
| 12 | Head motion-induced worsening | Does moving your head worsen your headache? |
| 13 | Mode of onset | Does it start suddenly like a thunderclap? |
| 14 | Location of pain | In which area of your head do you have a headache?  • Forehead  • Temple  • Back of the head  • Back of the neck  • Periocular  • Face  • All over the head  • Top of the head  • Behind the ear  • Side of the head  • Band-like |
| 15 | Nature of pain | How does your head feel like when you have a headache?  • Throbbing  • Pulsating  • Heart-beating  • Pounding  • Drumming  • Squeezing  • Splitting  • Heavy  • Tight band  • Dull ache  • Vague, cloudy  • Sharp  • Piercing  • Twinge  • Electric shock  • Burning  • Tingling  • Stabbing  • Thunderous  • Hammered like pain  • Explosive  • Jabbing |
| 16 | Headache attack right after waking up | When do you have a headache during the day?  • Right after waking up |
| 17 | Headache attack in the morning | • In the morning |
| 18 | Headache attack in the afternoon | • In the afternoon |
| 19 | Headache attack at night | • At night (during sleep) |
| 20 | Headache attack all day long | • All day long |
| 21 | Headache attack without specific pattern | • No specific pattern |
| 22 | Severity | Please rate the intensity of your most severe headache (out of 10). |
| 23 | Aggravation by physical activity | Does your headache worsen if you perform daily activities (such as walking or climbing the stairs)? |
| 24 | Avoidance by physical activity | Do you need to stop daily activities (such as walking or climbing the stairs) and take a rest when you have a headache? |
| 25 | Nausea and vomiting | If you have experienced the following symptoms during a headache attack, please mark.  • Nausea and/or vomiting |
| 26 | Ocular pain | • Eyes ache as if they are going to pop out |
| 27 | Photophobia | • Become sensitive to bright lights |
| 28 | Phonophobia | • Become sensitive to loud noises |
| 29 | Osmophobia | • Become sensitive to smell |
| 30 | Vertigo | • Dizzy as if you are spinning |
| 31 | General weakness | • You are not energetic generally |
| 32 | Blurred vision | • Eyesight is hazy/misty |
| 33 | Ear fullness/tinnitus | • Ear fullness and/or tinnitus |
| 34 | Hearing difficulty | • Difficulty in hearing |
| 35 | Diplopia | • Double vision |
| 36 | Allodynia | • Pain evoked by light touch or brushing |
| 37 | Cognitive complaint during headache attack | • Loss of memory |
| 38 | Inattention | • Inattention |
| 39 | Sensory aphasia | • Trouble understanding other’s speech |
| 40 | Dysarthria | • Slurred speech |
| 41 | Motor aphasia | • Trouble saying what was intended |
| 42 | Hemiparesis | • Hemibody paralysis |
| 43 | Loss of consciousness | • Loss of consciousness |
| 44 | Agitation | • Become restless/antsy |
| 45 | Lacrimation | • Tearing from only one eye |
| 46 | Rhinorrhea | • Watery nose from only one side |
| 47 | Nasal congestion | • Clogged nose from only one side |
| 48 | Facial swelling | • Facial swelling in only one side |
| 49 | Ptosis | • Drooped eyelid in only one side |
| 50 | Anhidrosis | • No sweats in only one side of the face |
| 51 | Conjunctival injection | • Red eye in only one side |
| 52 | Ear fullness | • Ear fullness in only one side |
| 53 | Visual aura | If you have any of the following symptoms (auras) before a headache, please choose from the below.  • Bright lights start to flash.  • Cracked in a zig-zag manner.  • Partial loss of vision.  • Smeared  • Vision is excessively bright.  • Sways like heat haze (heat shimmer). |
| 54 | Sensory aura | • Tingling or stings in one-sided hand/face  • Numbness in one-sided hand/face |
| 55 | Brainstem aura | • Dizzy as if spinning.  • Hear a ringing in the ears.  • Can’t hear well.  • Double vision.  • Can’t keep balance and stagger.  • Loss of consciousness. |
| 56 | Language aura | • Can’t fully express yourself  • Can’t understand others well.  • Slurred speech. |
| 57 | Motor aura | • Weakness in one-sided arms/legs. |
| 58 | Retinal aura | • One eye can’t see. (Partially or fully)  • One eye’s vision is distorted. |
| 59 | Hypertension | Do you have any illnesses listed below?  • Hypertension |
| 60 | Diabete mellitus | • Diabete mellitus |
| 61 | Hyperlipidemia | • Hyperlipidemia |
| 62 | Stroke | • Stroke |
| 63 | Heart disease | • Heart disease |
| 64 | Thyroid disease | • Thyroid disease |
| 65 | Allergy | • Allergy |
| 66 | Asthma | • Asthma |
| 67 | Insomnia | • Insomnia |
| 68 | Snoring | • Snoring |
| 69 | Head trauma | • Head trauma |
| 70 | Obstructive sleep apnea | • Obstructive sleep apnea |
| 71 | Temporomandibular disorder | • Temporomandibular disorder |
| 72 | Cervical Herniation of Inter-Vertebral Disc | • Cervical Herniation of Inter-Vertebral Disc |
| 73 | Fibromyalgia | • Fibromyalgia |
| 74 | Myofascial pain syndrome | • Myofascial pain syndrome |
| 75 | Caffeine | Do you usually take caffeine drinks such as coffee and tea? |

**Classification of secondary headache disorders.** We included secondary headache disorders as one of the target subtypes in the stacked XGBoost classifier. The addition of secondary headache disorders would lead to modeling six subtypes, 96 extra subjects in the training cohort, and 100 extra subjects in the test cohort. We trained the stacked classifier using the same method described in the main text for the six subtypes. The confusion matrix for the test cohort (Supplementary Table S3) shows degraded performance compared to our proposed modeling with five subtypes (Table 5). The main reason for the worsened performance was due to the low specificity of 0.1400 in secondary headache disorders. The secondary headache disorders are highly heterogenous and thus the classifier performance confirmed this heterogeneity.

**Supplementary Table S2**. Confusion matrix with secondary headache for the training cohort.

|  |  | Migraine | TTH | TAC | Epicranial headache | TCH | Secondary headache |
| --- | --- | --- | --- | --- | --- | --- | --- |
| Headache subtype | **Migraine** | **791** | 23 | 3 | 11 | 17 | 19 |
|  | **TTH** | 35 | **78** | 1 | 13 | 4 | 13 |
|  | **TAC** | 14 | 1 | **50** | 4 | 6 | 4 |
|  | **Epicranial headache** | 16 | 6 | 3 | **69** | 3 | 7 |
|  | **TCH** | 30 | 5 | 4 | 1 | **52** | 3 |
|  | **Secondary headache** | 33 | 8 | 4 | 8 | 12 | **31** |

TTH, Tension-type headache; TAC, Trigeminal autonomic cephalalgia; TCH, Thunderclap headache.

**Supplementary Table S3**. Confusion matrix with secondary headache for the test cohort.

|  |  | Migraine | TTH | TAC | Epicranial headache | TCH | Secondary headache |
| --- | --- | --- | --- | --- | --- | --- | --- |
| Headache subtype | **Migraine** | **546** | 5 | 8 | 6 | 22 | 13 |
|  | **TTH** | 21 | **40** | 0 | 12 | 4 | 14 |
|  | **TAC** | 17 | 1 | **25** | 2 | 9 | 3 |
|  | **Epicranial headache** | 7 | 6 | 2 | **33** | 6 | 7 |
|  | **TCH** | 16 | 2 | 4 | 7 | **35** | 3 |
|  | **Secondary headache** | 51 | 11 | 3 | 15 | 6 | **14** |

TTH, Tension-type headache; TAC, Trigeminal autonomic cephalalgia; TCH, Thunderclap headache.
